# Professor C. L. Ford’s Questions

**Published:** 1890-02

**Authors:** 


### Prof. C. L. Ford's Questions.

Below is given a list of questions upon the structure of the teeth which will somewhat illustrate his method of procedure in his course of instruction to the dental classes, in the Dental Department, of the University of Michigan; it will not only serve this purpose, but may lead others to study the points involved in these questions. They are worthy of special attention:

QUESTIONS ON TEETH.

1. Name all the tissues found in the human tooth.
2. What part of a tooth does each of these form?
3. What is the hardest substance in the human body?
4. What structural elements are found in enamel?
5. How are enamel prisms or rods arranged?
6. Why are they perpendicular on the masticating surface?
7. How large are the enamel prisms, and how shaped?
8. Are they of uniform size in all parts of a tooth?
9. In what way are these rods so firmly united?
10. What proportion of animal and mineral material?
11. What, and where are the transverse striæ of Retzius?
12. Does a tooth possess power to repair the enamel?
13. Does the enamel possess ordinary sensibility?
14. In what way is enamel united to dentine?
15. In development of teeth which tissue is formed first?
16. What tissue of teeth is least in quality, and where is it?
17. What part of a tooth does this substance form?
18. By what different names is this tissue known?
19. What other tissue of the body does it resemble?
20. State in what respect this resemblance consists.
21. What is named as present in normal cement?
22. What is sometimes found when increased in quantity?
23. By what name is increase in cement known?
24. Is healthy cement endowed with much sensibility?
25. What tissue makes most of the tooth substance?
26. Is the dentine everywhere covered? If so, how?
27. What part of a tooth is called its ivory, and why?

28. What proportion of animal and mineral matter in dentine?
29. What is meant by dentine cartilage, and how obtained?
30. What is understood by the matrix of a tooth?
31. What is meant by inter-tubular substance?
32. Is this substance known by any other names?
33. What is the nature or character of this substance?
34. What canals or channels are seen in dentine?
35. Describe their commencement and termination.
36. What is their direction through the dentine?
37. How large are these channels in the dentine?
38. Are they equally abundant in all parts of it?
39. In what manner do they branch and anastomose?
40. By what different names are these channels known?
41. Do the dental tubuli join the canaliculi in cement?
42. Do they communicate with spaces in enamel?
43. What immediately surrounds the dental tubuli?
44. What is the Sheath of Neumann, and where found?
45. How are the dental tubuli occupied in life?
46. What is the source of such substance or fibrils?
47. Why do they receive the name usually given?
48. What is meant by the dental sheath, and where is it?
49. Describe the character and property of this sheath.
50. What tissue of the teeth has the greatest sensibility?
51. Where is the sensitiveness of dentine greatest?
52. Is dentine sensitive after the pulp is destroyed?
53. What is meant by contour lines, and where seen?
54. What, and where in tooth is the granular layer?
55. What, and where are the inter-globular spaces?
56. Are these appearances found in all teeth?
57. Are they the result of imperfect development?
58. What is the tooth pulp, and where is it situated?
59. Of what is it composed, and its usual color?
60. How do nerves and blood vessels reach the pulp?
61. What other constituents assist to form the pulp?
62. Of what structure is the pulp the remains?
63. What is meant by the membrane eboris; where?
64. Where are found the odontoblast cells?
65. Describe these cells, and their processes?
66. Where, and what are Tomes fibres, or tooth fibrils?
67. With what are they connected in the pulp?
68. What purpose do they serve in the teeth?
69. Are Tomes fibres to be considered as nerves?
70. What is Nesmyth's membrane and where found?
71. State concisely what you learn concerning it?

